# Digital Twin Fundamentals of mRNA *In Vitro* Transcription in Variable Scale Toward Autonomous Operation

**DOI:** 10.1021/acsomega.3c08732

**Published:** 2024-02-05

**Authors:** Alina Hengelbrock, Axel Schmidt, Jochen Strube

**Affiliations:** Institute for Separation and Process Technology, Clausthal University of Technology, Clausthal-Zellerfeld 38678, Germany

## Abstract

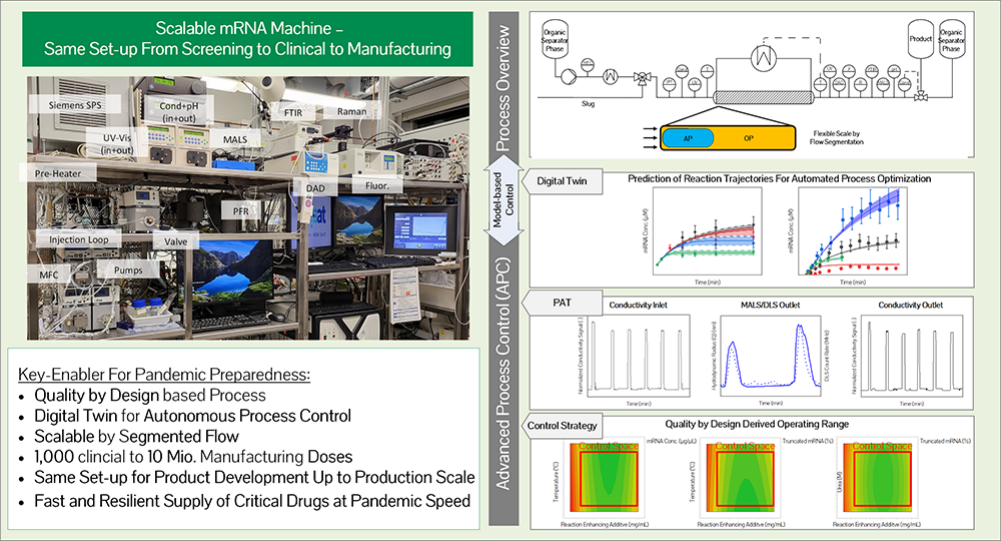

The COVID-19 pandemic caused the rapid development of mRNA (messenger ribonucleic acid) vaccines and new RNA-based therapeutic methods. However, the approval rate for candidates has the potential to be increased, with a significant number failing so far due to efficacy, safety, and manufacturing deficiencies, hindering equitable vaccine distribution during pandemics. This study focuses on optimizing the production of mRNA, a critical component of mRNA-based vaccines, using a scalable machine by investigating the key mechanisms of mRNA *in vitro* transcription. First, kinetic parameters for the mRNA production process were determined. The validity of the determination and the robustness of the model are demonstrated by predicting different reactions with and without substrate limitations as well as different transcripts. The optimized reaction conditions, including temperature, urea concentration, and concentration of reaction-enhancing additives, resulted in a 55% increase in mRNA yield with a 33% reduction in truncated mRNA. Additionally, the feasibility of a segmented flow approach allowed for high-throughput screening (HTS), enabling the production of 20 vaccine candidates within a short time frame, representing a 10-fold increase in productivity, compared to nonsegmented reactions limited by the residence time in the plug flow reactor. The findings presented for the first time here contribute to the development of a fully continuous and efficient manufacturing process for mRNA and other cell and gene therapy drugs/vaccine candidates as presented in our previous work, which discussed the integration of process analytical technologies and predictive process models in a Biopharma 4.0 facility to enable the production of clinical and large-scale doses, ensuring a rapid and resilient supply of critical therapeutics. The results in this study especially highlight that the same machine and equipment can be used for screening and manufacturing different drug candidates in continuous operation. By streamlining production and adhering to quality standards, this approach enhances the industry’s ability to respond swiftly to pandemics and public health emergencies, addressing the urgent need for accessible and effective vaccines.

## 1 Introduction

The biopharmaceutical industry is constantly seeking new and innovative ways to reduce the time-to-market and risk associated with the development of new therapies and vaccines.^1^ This need for speed is particularly pressing in the face of pandemics and other public health emergencies, where swift action is critical to prevent further harm to the population.^2^ In these situations, regulatory agencies such as the US Food and Drug Administration (FDA) and the European Medicines Agency (EMA) implement accelerated review processes to facilitate the development and approval of treatments and vaccines.^3^

However, the likelihood of approval (LOA) for drug candidates from phase I in 2006 to 2015 is known to be only 10% over all indications.^4^ Scientific and regulatory reasons for delay and denial of FDA approval of initial applications for new drugs were investigated,^5^ where it was found that in 2000 to 2012, of 302 new molecular entities (NMEs) submissions, 50% failed the first approval cycle. A total of 84.8% failed due to efficacy and/or safety deficiencies. The remaining 15.2% failed due to CMC (chemistry, manufacturing, and controls) and/or labeling deficiencies. From the 50% first-cycle failures, 53% were never approved during the study. Although, in general, success rates for vaccines are higher than for drugs,^6^ each failed candidate leads to more hurdles for vaccine equity in pandemic scenarios. The remarkable speed at which vaccines in times of need, under political and societal pressure, as seen with BNT162b2 and mRNA-1273, got market authorization serves as an example of already reachable time reductions for development and approval.^7^

Reflecting the lessons learned from drug and vaccine development over the past 20 years, especially from COVID-19, ways to increase speed and flexibility for candidate screening and development while minimizing risks and deficiencies related to efficacy, safety, and CMC are urgently needed. An approach to drug and vaccine development, which involves using the same equipment and processes for both preclinical studies and commercial production, could help bring new drugs to market faster and more efficiently.^8^ Ensuring that 1000 doses in the (pre)clinical phase and up to 10 mil. doses in the manufacturing phase can be performed on the same process and machine setup will require the combination of scale approaches, such as segmented slug flow (where small doses are produced by processing few slugs) and continuous operation and automation concepts,^9^ enabled by process analytical technology (PAT)^10^ and digital twins (DTs),^11^ which are able to handle varying process volumes.

This work will show the investigation of key mechanisms of mRNA *in vitro* transcription (IVT) as the first step in mRNA-based vaccine manufacturing on the proposed scalable machine, including the potential for accelerated candidate and process parameter optimization, to enable quick, scalable, risk-reduced, and resilient supply of critical therapeutics at pandemic speeds.

## 2 Fundamentals and State of the Art

### 2.1 Mechanics of *In Vitro* Transcription

*In vitro* transcription (IVT) is an enzymatically catalyzed polymerization reaction. Typically, T7, SP6 or T3 polymerases are used, with the T7 RNA polymerase being the main representative.^12,13^ It has a high enzymatic activity; moreover, modified nucleotides can be used as a substrate.^14^

In the following reaction scheme, IVT catalyzed by a T7 RNA polymerase is shown.^13^ IVT is initiated via the initiation reaction (eqs 1–5) followed by elongation (eq 6) and finally termination of the reaction (eq 13).^13,15,16^ Here, *K*_i_ describes reversible reactions. Irreversible reactions are expressed in terms of rate constants kI, kE, and kT. In initiation, a GTP (guanosine triphosphate) molecule binds randomly to the promoter sequence (D) on the template DNA (DNA) and is completed when the reversible enzyme-DNA-RNA complex is formed (E·D·Mj). This is followed by elongation (eq 6), in which one of the nucleotides (NTPs) ATP (adenosine triphosphate), CTP (cytidine triphosphate), UTP (uridine triphosphate), or GTP, depending on the template DNA sequence, is irreversibly bound to the mRNA, and a pyrophosphate molecule is cleaved off.^12,13,17^

These two key reaction steps of IVT cannot take place without the cofactor magnesium since the T7 polymerase is a magnesium-dependent enzyme. In addition to catalyzing the initiation and elongation reactions, the magnesium ions also form complexes with the nucleotides, which form phosphodiester bonds with the mRNA chain, and the pyrophosphate is cleaved.^12,17−20^

The nucleotides can bind to the free enzyme, the promoter-enzyme complex, or the transcription complex (eqs 7–11). The cleaved pyrophosphate can transiently bind to the nucleotide binding site of the free enzyme or to the enzyme-DNA-RNA complex (eq 12). These multiple binding possibilities result in competition for the binding sites and thus competitive inhibition of the enzyme.^13,21,22^ Furthermore, the cleaved pyrophosphate ion can form insoluble complexes with magnesium ions and precipitate.^12,23^ To prevent the inhibition as well as the formation of the insoluble pyrophosphate-magnesium complexes, pyrophosphatase is usually added, which hydrolyzes the pyrophosphate into inorganic phosphate.^12,21,22,24^ Once the mRNA reaches its final length, the enzyme-DNA-RNA complex disintegrates, releasing the mRNA (Mn), enzyme, and template DNA.^13^ The termination kinetics is not relevant if runoff transcriptions are performed.

The overall framework shown in eqs 1 to 13 was introduced by Arnold et al.^13^

Initiation:
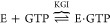
1
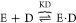
2
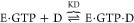
3
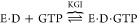
4
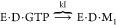
5

Elongation:
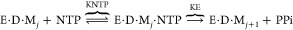
6

Competitive inhibition:
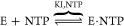
7
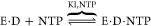
8
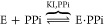
9
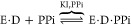
10
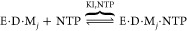
11
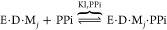
12

Termination:
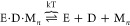
13

In many studies, nucleotide concentrations, magnesium concentrations, and their interactions with each other have already been reported to be the most significant factors in IVT.^8,25−28^ Some of these will give specific concentrations of magnesium that vary over a wide range from 12 to 60 mM.^12,20,28−32^ Others also indicate optimal ratios of NTP to magnesium ions.^32^ Furthermore, the counterion to magnesium ions plays a role, as magnesium acetate can be used at higher concentrations, unlike magnesium chloride.^32,33^

During *in vitro* transcription, in addition to single-stranded RNA (ssRNA), the formation of double-stranded RNA (dsRNA) can also occur. In the literature, two major mechanisms of dsRNA formation while performing IVT with the T7 RNA polymerase are frequently mentioned. In the self-priming mode, the RNA polymerase uses the RNA as a template and hybridizes the 3′ end of a full-length RNA with itself to form a duplex region.^34−38^ This duplex region is generated in the antisense mode by annealing an abortive 5- to 11-nt RNA with a different transcript.^34,37−40^ It was demonstrated that by adding chaotropic substances such as urea or formamide, a mild chaotropic environment can be created in which the polymerase is still functional, but weak unwanted RNA hybridization is disrupted.^34^

In addition to dsRNA, short RNA fragments from abortive cycles during initiation or from hydrolytic degradation of full-length mRNA represent another undesired impurity in *in vitro* transcription.^41−44^ During the initiation reaction, a molten bubble forms in the elongation complex. This migrates with the enzyme along the template DNA during transcription and is typically very stable, as it is a ternary complex. However, at the beginning of the transcription reaction, this complex is relatively unstable, resulting in the release of a short number of RNA transcripts. These 2–8 base short RNA molecules restart transcription. This process is also called the abortive cycle.^44−48^ Although the abortive cycle is broken after approximately 10–14 bases, the RNA polymerase loses sequence-specific contacts with the promoter sequence in the process. Thus, a processive elongation complex is formed in which the RNA chain is extended independently from the sequence on the template DNA.^44,49−51^

To enhance the reaction rate and yield, additives can be employed;^52^ these additives include water,^53^ water mimics,^54^ organic bases,^55^ cosolvents,^56^ crown ethers,^57^ salts,^58^ and some particular molecular additives, e.g. (but not limited to), polysorbates,^59^ betaine,^60^ alkylpolyglycosides,^61^ and sucrose fatty acid ester.^62^ Here, we investigate the effect of additives of types of low-chaotropic or amphoteric agents to modify miscibility in a range of few to thousands of molecular daltons.^63^

### 2.2 Analytics and mRNA Quality Aspects

After IVT, different process-related and product-related impurities will be present.

Process-related impurities originate from input materials, including enzymes (T7 RNA polymerase, RNase inhibitor, and pyrophosphatase), (modified) nucleotides (ATP, CTP, GTP, and UTP), cap analogues (e.g., ARCA or CleanCap), and template DNA (PCR or plasmids).

Product-related impurities are truncated mRNA, also termed fragments as well as double stranded mRNA (dsRNA), and are typically quantified as drug substance (DS) critical quality attributes (CQAs). Other CQAs include the poly(A)tail length, 5′cap analysis, and mRNA sequence.

To monitor IVT progress as well as to quantify process- and product-related impurities, different chromatographic separation media are available. All nucleotides and cap analogs can be baseline separated by strong anion exchange (AEX), e.g., on a Thermo Fisher DNA Pac PA200.^64,65^ Product-related impurities (truncated mRNA and dsRNA) are typically resolved by ion-pair reversed-phase chromatography (IP-RP), e.g., on a Thermo Fisher DNA Pac RP.^66^ The amount of mRNA with the polyadenylated tail can be quantified by affinity media functionalized with oligo dT ligands.^67^ To increase the analytical speed, application of monolithic media is often reported in the literature, with the mixed-mode type (BIA Separations CIMAC PrimaS, bimodal AEX+HIC)^68^ being proposed for quantification of cap analogs, nucleotides, template DNA, and mRNA.^69^ The total monolith method length is 8 min, compared to 20 min on bead-based strong AEX, which has a higher resolution in regard to single nucleotide separation, whereas UTP and CTP coelute on the monolith (though quantities can be estimated by the UV absorbance ratio at 260/280 nm).^69^ Here, we chose a combination of AEX and IP-RP chromatography for a better resolution necessary to determine specific IVT reaction kinetics. The speed advantage of the monolithic media might be preferable if at-line analytics are part of the control strategy, though, to realize continuous, automized manufacturing based on DTs, real-time information enabled by PAT is necessary anyway.

**Figure 1:**
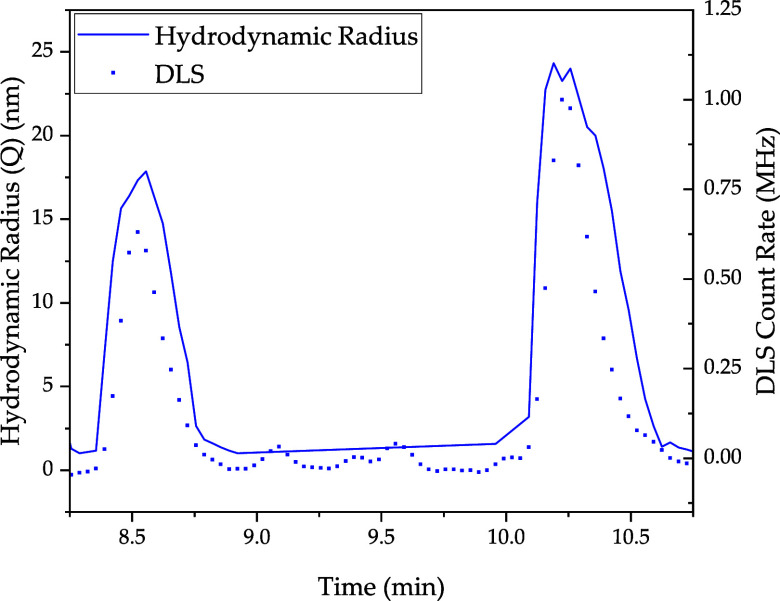
Ion-pair reversed-phase chromatogram of the mRNA *in vitro* transcription product with smaller transcripts corresponding to the first peak and the full-length transcript corresponding to the second peak.^8^

An important information obtained by IP-RP analytics is the evaluation of mRNA integrity and purity regarding the presence of truncated mRNA and dsRNA. Already in 2021, the public assessment report for BNT126b2 by the EMA for initial authorization mentions the observed dependency of limiting ATP and CTP amounts in regard to mRNA integrity (e.g., the amount of truncated mRNA) and states that further data are to be evaluated as the first specific obligation (SO1), especially as the presence of truncated mRNA has been approved in the final DS. However, the report also states that expressed proteins by truncated/fragmented species are unlikely due to their lower stability and poor translation efficiency.^70^ Recently, Patel et al. analyzed the truncated/fragmented species in BNT126b2, observed as an early eluting peak in IP-RP, by capillary gel electrophoresis and found that they are indeed only present in the first peak, while the second main peak only represents the functional, full-length ss-mRNA.^42^ These results are in line with IP-RP analytics in combination with MALS-DLS (see Figure 1) detection, which showed two major peaks; the first one corresponds to smaller mRNA truncates/fragments (approximately 15 nm), and the second one corresponds to the expected full-length ssRNA (approximately 23 nm). This also confirmed the initial hypothesis that since most of the fragmented species include the 5′cap but lack the poly(A) tail, their formation is mostly caused by premature IVT reaction termination.^42^ The other product-related impurity, dsRNA, is found in the tailing of the main product peak in IP-RP.^66,71^

### 2.3 Feeding and Optimization Strategies

As batch IVT is still state-of-the-art in mRNA vaccine manufacturing, all reactants are usually combined in the beginning; hence, at least one of these components is posed to be limiting at the end of the transcription reaction. The result of limiting nucleotides, as discussed above, can be the formation of truncated mRNA and even poly(A) tail variations.

Moderna filed a patent in 2020 for fed-batch IVT processes.^72^ The patent discloses methods that describe aspects for continuous or bolus feeding of nucleotides according to their rate of consumption in terms of balancing relative molar ratios of each nucleotide to maximize the use of reactants and/or to alter attributes of the mRNA product so that no nucleotide is rate-limiting during the IVT reaction. In one embodiment of the method, consumption rates during batch IVT are first recorded to derive later a feeding strategy for the fed-batch process; however, strategies based on ongoing consumption rate measurement are also described. Compared to a reference batch IVT reaction, they found a 1.8-fold higher yield of mRNA. Recently, BioNTech published results demonstrating that controlled low UTP levels lead to decreased dsRNA formation while maintaining mRNA yield and integrity.^73^

Pregeljc et al. also investigated the IVT yield dependence on different process and feeding strategies.^12^ They tested not only for nucleotide consumption but also for bolus addition of Mg^2+^ as its concentration effect on the IVT reaction rate and mRNA yield is known to vary depending on the construct length and sequence. Their results lead to the conclusion that the addition of nucleotides alone is not the key factor for increased reaction rates and higher yields. Rather, the combined feeding with Mg^2+^ revealed it as the rate-limiting component. Ultimately, applying this feeding strategy, they reached a 4.5-fold increase in mRNA yield, compared to their reference batch IVT.

The optimization approaches for fed-batch IVT shown in the literature highlight the potential benefit that can be achieved by continuous IVT. Rosa et al. proposed a microfluidic concept with subsequent downstream process steps.^74^ The ability to not only redirect enzymes but also nucleotides and cost-intensive cap analogs at key positions of the PFR (plug flow reactor) can further increase the productivity gains already obtained by continuous IVT even more in terms of COG (cost of goods) and GWP (global warming potential),^75^ therefore enabling economic-competitive and more sustainable production.^76^

### 2.4 Modeling and Simulation

Predictive process models that are capable to accurately describe the effects of fluid dynamics, kinetics, and thermodynamic equilibrium are ultimately needed, also often referred to as mechanistic models,^77−79^ which are essential for early process development and optimization as well as a basis for DT-enabled continuous, automized manufacturing. Such models are rare in the literature.

Arnold et al. developed a model that provided the functional dependence of T7 RNAP kinetics based on reactant concentrations and transcript specifics such as the transcript sequence and length.^13^ The focus on models discussed in the literature is to be seen within the operating regimes and design spaces of the IVT reaction that they investigated.^80^ Akama et al.^81^ for example applied a Michaelis–Menten-type equation to the transcription reaction and a semiempirical equation describing the correlation between the induction period and the supersaturation ratio to the precipitation formation, respectively. The authors saw the application of their model in system-level analysis of bone formation in living organisms.

Da Gama and Petridis applied for technoeconomic analysis a mathematical model relying on the macroscopic stoichiometric IVT reaction equation.^82^ Ouranidis et al. applied a Michaelis–Menten equation-based model that also included equipment- and process-specific parameters such as the required mixing time and agitation power input.^83^ A mechanistic model was part of the QbD (quality-by-design) study on mRNA production by van de Berg et al.^84^ The differential-algebraic equation system described the interdependency of mRNA concentration based on kinetic terms for transcription, degradation, and precipitation considering the mass balance and equilibrium conditions. They also applied the model for sensitivity analysis and concluded that it performed well in comparison to conventional statistical model approaches. Remarkably, they found that the phenomena of enzyme degradation and Mg_2_PPi precipitation could be ignored; hence, they set rate factors to 0, as they did not improve model performance.

This highlights again that “operating regimes and design spaces of the IVT reaction” are fundamental to knowing which effects are of importance and which are not.^80^ While the precipitation mechanism might affect IVT performance in certain scenarios, given the current research by key industry players,^72,73^ it is obvious that optimization of individual NTP concentration and feeding is currently one of the main focuses to advance IVT manufacturing. This can only be described by a model approach that considers the NTP individually, like the Arnold et al. model.^13^ Another process model based on the work of Arnold et al., which was based on Michaelis–Menten kinetics as well as thermodynamic equilibrium, was augmented to include fluid dynamics behavior in different reactor regimes and studied extensively in regard to the process strategy (batch reactor, continuously stirred tank reactor, and plug flow reactor) following a QbD-based methodology, including risk assessment and sensitivity studies and design space determination^85^ and recently as DT for process automation and control studies.^9^

As described in Sections 2.2 and 2.3, the characterization of limiting NTP concentrations on quality aspects^70^ and the optimization of feeding strategies regarding individual NTPs are the subject of current research and development.^72^*K*_m_ values are fundamental to the Arnold et al. model. The Michaelis–Menten constants become relevant mainly when the substrate concentrations fall below the respective threshold of <100 times *K*_m_.^86^ This is more likely the case as the reaction progresses, which is why it becomes especially important in the implementation of appropriate feeding strategies.

## 3 Materials and Methods

### 3.1 Preparation of the Linearized pDNA Template

In order for the T7 RNA polymerase to transcribe the desired mRNA, a linearized DNA template is required. The BNT162b2 template was purchased from GenScript (GenScript Biotech Corporation, Piscataway, NJ, US). The pDNA was linearized with the restriction enzyme EcoRI (Thermo Scientific, Waltham, MA, USA) so that the polymerase can read it.^87^ This was followed by an ultrafiltration and diafiltration step with the help of a 100 kDa membrane (Vivaspin 2, Sartorius AG, Göttingen, Germany) to adjust the concentration and to buffer the pDNA in 10 mM Tris-HCl at pH 8 to stabilize the pDNA.

### 3.2 Analytical Methods

#### 3.2.1 Agarose Gel Electrophoresis

Agarose gel gives the mRNA titer in total as well as qualitative homogeneity and length. The gel consisted of 1.2% agarose, 1× TAE buffer, and ethidium bromide; the 1× TAE buffer was also used as the running buffer. Before loading the gel, the samples were first denatured by adding formamide (60% v/v final concentration), and denaturation of the samples was done for 5 min at 65 °C.^88^ The samples were diluted to 1:10, 1:20, 1:100, and 1:200 to prevent overloading of the gel.^89,90^ Electrophoresis was performed at 120 V for 60 min.

#### 3.2.2 AEX Chromatography

High-pressure liquid-chromatography (HPLC) analytics were adapted from Kanavarioti^65^ and performed on an Agilent 1100 system (Agilent Technologies, Inc., CA, USA). The column was a DNAPac PA200 (Thermo Scientific, Waltham, MA, USA). A gradient for 16 min from 0% mobile phase A (MPA_AEX_; 10 mM NaOH, pH 12.0) to 95% mobile phase B (MPB_AEX_; 10 mM NaOH, 1.5 M NaCl, pH 12.0) at a flow rate of 0.9 mL/min was applied followed by a re-equilibration to 100% MPA_AEX_ for 4 min. Before injecting the samples, a dilution with MPA_AEX_ was done to stay within calibrated mass range.

#### 3.2.3 IP-RP Chromatography

The determination of truncated and intact mRNA was performed using IP-RP chromatography. A DNAPac RP column (Thermo Scientific, Waltham, MA, USA) with a guard column was used. The mobile phases were 100 mM TAE buffer at pH 7 (MPA_RP_) and 100 mM TAE with 25% acetonitrile at pH 7 (MPB_RP_). The method was adapted from the literature.^28,91^ One of the main discoveries of the inventors of the method was that by using unconventional ion-pairing agents such as Tris, polyA tail length-based separation from complex mixtures is possible in the absence of classical ion-pairing agents.^91^ Before loading the samples onto the column, the samples were diluted with mobile phase A (MPA_RP_) to get in the calibrated mass range. The relative amount of truncated mRNA was calculated by relating the peak area of truncated mRNA to the sum of the peak areas of truncated and intact mRNA.

### 3.3 *In Vitro* Transcription and Model Framework

*In vitro* transcription was performed batchwise for the determination of the enzymatic kinetic parameters and continuously in a plug flow reactor for high-throughput screening (HTS) of reaction parameters.

The reaction buffer was composed of 50 mM Tris-HCl, 10 mM DTT, 0.002% Triton, 1 U/μL RNase inhibitor, 0.002 U/μL pyrophosphatase, 8 U/μL T7 polymerase, and 0.05 μg/μL template. These factors were used in *in vitro* transcription at the concentrations frequently used in the literature.^28,31,89,90^ They were not varied because no optimization potential is known for them. Meanwhile, the NTP and magnesium acetate concentrations as well as the pH value and temperature had an impact on the mRNA yield. These factors were optimized previously for the system used in this study and amount to 10 mM NTP, 50 mM magnesium acetate, a pH value of 7, and a temperature of 37 °C.^8^

To determine the characteristic parameters of enzymatic reactions, the Michaelis–Menten constant (*K*_M_) and the maximum reaction rate (*v*_max_), the substrate concentration of one nucleotide was varied, whereas the other nucleotides were added in excess, and samples were taken at different time points and quenched with 5 mM EDTA. Since the reaction generates pyrophosphate, which is an inhibitor of *in vitro* transcription (see the reaction mechanism in Section 2.1), yeast inorganic pyrophosphatase (New England Biolabs, Inc., Ipswich, MA, US) was added to the reaction mix, which hydrolyzes the inorganic pyrophosphate to inorganic phosphate.^24^ In addition, the optimized reaction conditions for the mRNA transcribed in this study, described in detail in the literature, were adapted so that the varied nucleotide was the only limiting factor. The substrate concentrations used were 10, 7.5, 5, and 2.5 mM.

From the time courses of the reactions for the different concentration levels, the initial reaction rates were determined. This represents the rate at the beginning of the reaction, where the reaction rate is still increasing linearly. Arnold et al. described an advanced Michaelis–Menten kinetics model to describe the reaction velocity of the *in vitro* transcription:^13^
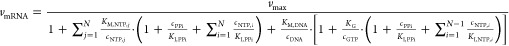
14

The advanced Michaelis–Menten equation describes the influence of the nucleotide concentrations (*c*_NTP_), the promoter (), and the competitive inhibition by the NTPs () and the pyrophosphate ().  and  represent the Michaelis–Menten constants of the nucleotides and the promoter, respectively.  is the dissociation constant for initial GTP binding, and  describes the concentration of guanosine triphosphate. The initiation process of IVT is described by the equation in the square brackets in the denominator of the first fraction.^13^

Since all nucleotides, except the nucleotide under investigation, are present in excess and the addition of pyrophosphatase eliminates inhibiting pyrophosphate in the initial reaction phase, the equation describing the reaction rate is simplified to the simple Michaelis–Menten kinetics.^13,86^ This equation applies only to the determination of the initial reaction rates for the determination of the kinetic parameters and not to the entire reaction process.  represents an apparent , which includes possible competitive inhibition.^86^ When the apparent Michaelis–Menten constants are used in eq 14, the competitive inhibition terms must not be included.
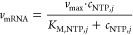
15

To simplify the determination of the kinetic parameters, the initial reaction rates and the substrate concentrations used were plotted as a Lineweaver–Burk, Hanes–Woolf, and Eadie–Hofstee diagram, and a linear regression was performed. Additionally, nonlinear regression of the Michaelis–Menten plot was performed.

The model for simulating the experimental data was first developed by Arnold et al.^13^ and applied for control studies in a previously published study.^9^ The change in mass over time, which is either produced or consumed in a particular reaction, is described by *v* according to the kinetic equation. The consumption of the produced pyrophosphate by the supplemented pyrophosphatase is described by a simple Michaelis–Menten equation:^80^
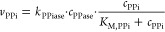
16with *k*_PPiase_ as the rate constant of the pyrophosphatase, *c*_PPase_ as the volume-based enzyme activity, and *K*_M,PPi_ as the Michaelis–Menten constant of pyrophosphate. The rate constant of the pyrophosphatase *k*_PPiase_ and the Michaelis–Menten constant of pyrophosphate were obtained from the literature.^92,93^

The changes in product and substrate concentrations over time were calculated by^13^
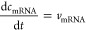
17
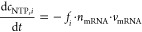
18
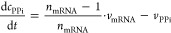
19where *f*_*i*_ is the relative portion of the base contained in the mRNA and *n*_mRNA_ is the transcript length of the mRNA.

**Figure 2:**
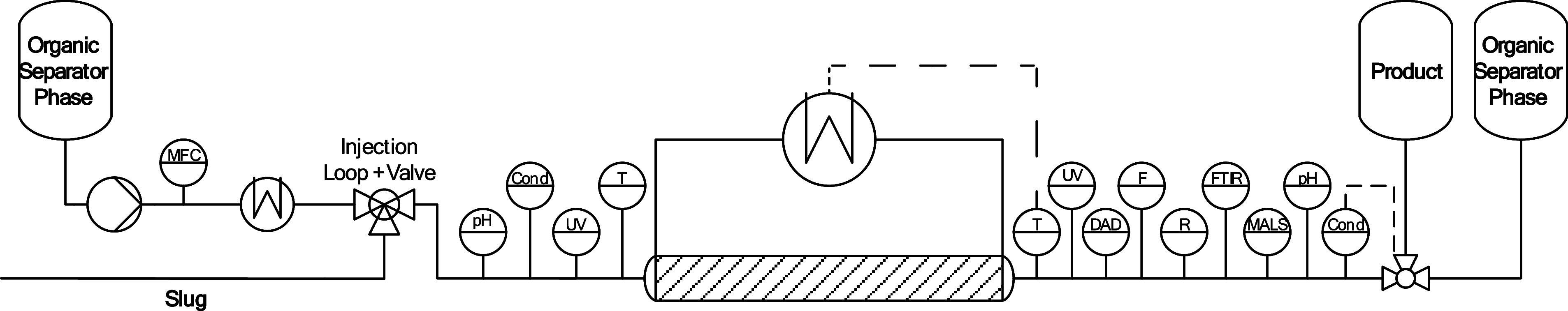
Setup of the test unit in which the segmented slugs are generated, detected, and fractionated by the resulting detection signal.^8^

The continuous production was performed by generating a segmented flow in a plug flow reactor with a 1/16 in. diameter and a length of approximately 18 m. The feasibility of the slug generation was already demonstrated.^8^ The reaction mixture formed the slug phase, and oleic acid was used as the slug generating phase. The experimental setup is shown in the flow diagram in Figure 2.

The transcription buffer and oleic acid were stored in tanks, from which the desired phase was pumped into the PFR. The fluid passed first a switch valve and a mass flow controller to monitor the actual volumetric flow rate and density and then passed a UV detector followed by a conductivity detector. At the exit of the PFR, the UV signal was captured first followed by the conductivity signal. With the downstream valve, the product fraction can be fractionated signal-based. The chosen wavelength for the UV detector was 200 nm since this is the wavelength at which the absorption maximum of oleic acid is located.^94^ The experiments of the experimental plan were performed by first filling the tubular reactor and tubing with oleic acid and then applying several slugs one after the other using the switching valve. The slug was displaced from the oil phase by switching the valve again. To ensure a required residence time of 2–3 h for the apparatus dimensions, the volume flow was set to 0.2 mL/min.

Since supplementation of urea has already been shown in the literature to reduce dsRNA during *in vitro* transcription,^34^ urea was supplemented during the continuous *in vitro* transcription.^8^ However, this resulted in a decrease in yield and a significant increase in truncated mRNA in continuous production. Consequently, a full factorial experimental design comprising 15 experimental points including three center points was designed to optimize the reaction conditions in terms of yield and the proportion of fully formed mRNA. For this purpose, the temperature, urea concentration, and reaction-enhancing additives were varied. Piao et al. observed that total shutdown of the polymerase can occur with the addition of 1.2 M urea at a temperature of 40 °C. In contrast, a low temperature of 37 °C and a urea concentration of 0.8 M were identified as the optimum yield and proportion of dsRNA, with higher yields obtained at 0.4 M urea.^34^ Consequently, in this study, to optimize continuous IVT, the temperature was varied in the range of 37 to 40 °C and the urea concentration in the range of 0.4–1.2 M. The selected concentration range for the reaction-enhancing additives was between 0.1 and 1 mg/mL.

## 4 Results

### 4.1 Calibration of the Chromatographic Analytics

**Figure 3:**
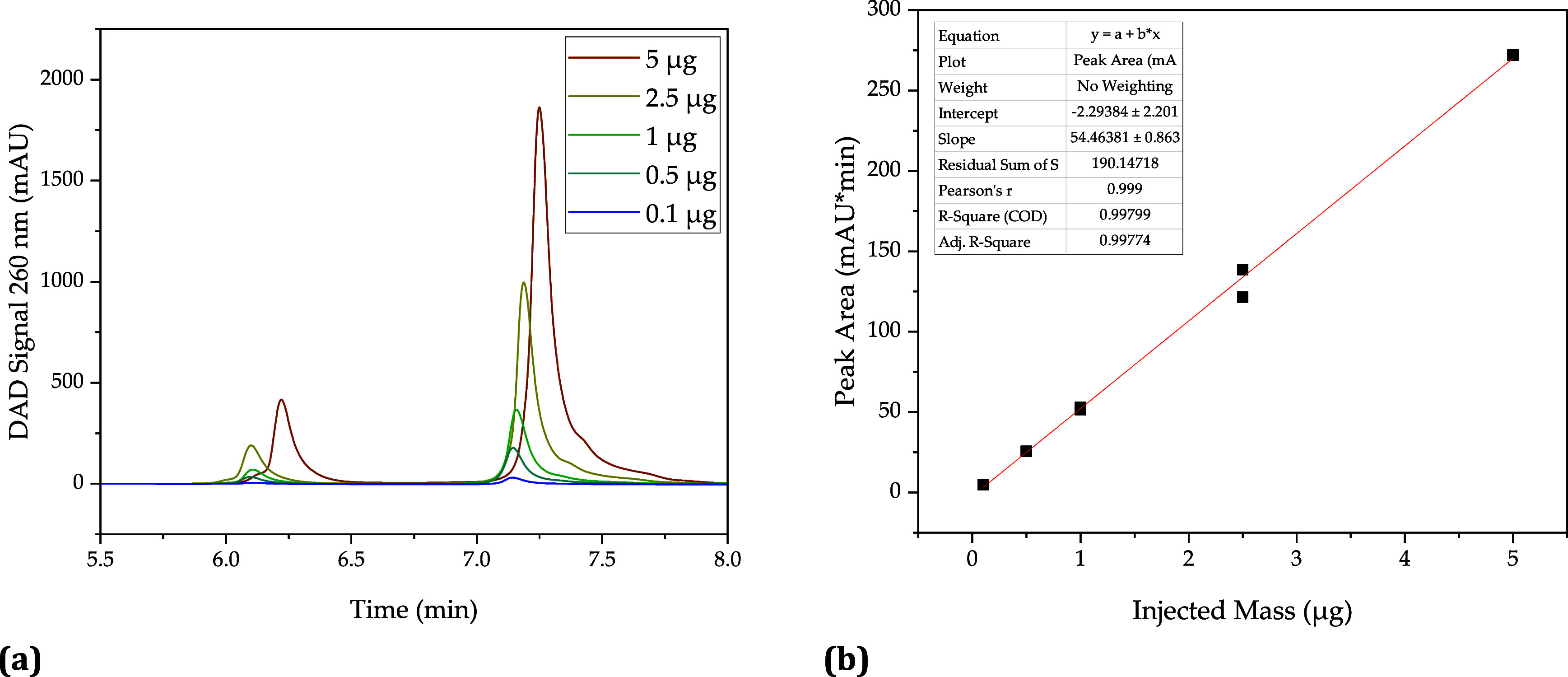
Calibration of the mRNA concentration measured by ion-pair reversed-phase chromatography. Chromatogram with increasing injection mass (a) and calibration line (b).

IP-RP chromatography is used and calibrated to determine the amount of truncated mRNA. CleanCap Cas9 mRNA (TriLink Bio Technologies, San Diego, CA, US) is used for calibration, as its size of 4521 nucleotides corresponds approximately to the size of the BNT162b2 transcript produced in this study with a size of 4284 nucleotides. Amounts of 0.1, 0.5, 1, 2.5, and 5 μg were injected, and duplicate determinations were performed in each case. The chromatograms and the regression curve of the peak areas are shown in Figure 3. When injecting larger masses of mRNA, a slight shift of the main peak at minute 7.2 to the right can be seen (Figure 3 a). This is most prominent at the 5 μg injection, so the calibrated range up to a 2.5 μg injection mass should be preferred for analysis. The *R*^2^ of the calibration line is very high with a value of 0.998, so that the linear relationship of the injected mRNA mass and peak area is given (cf. Figure 3b). In addition, hardly any deviations can be detected in the double determination, so that very high reproducibility of the measurements can be concluded.

**Figure 4:**
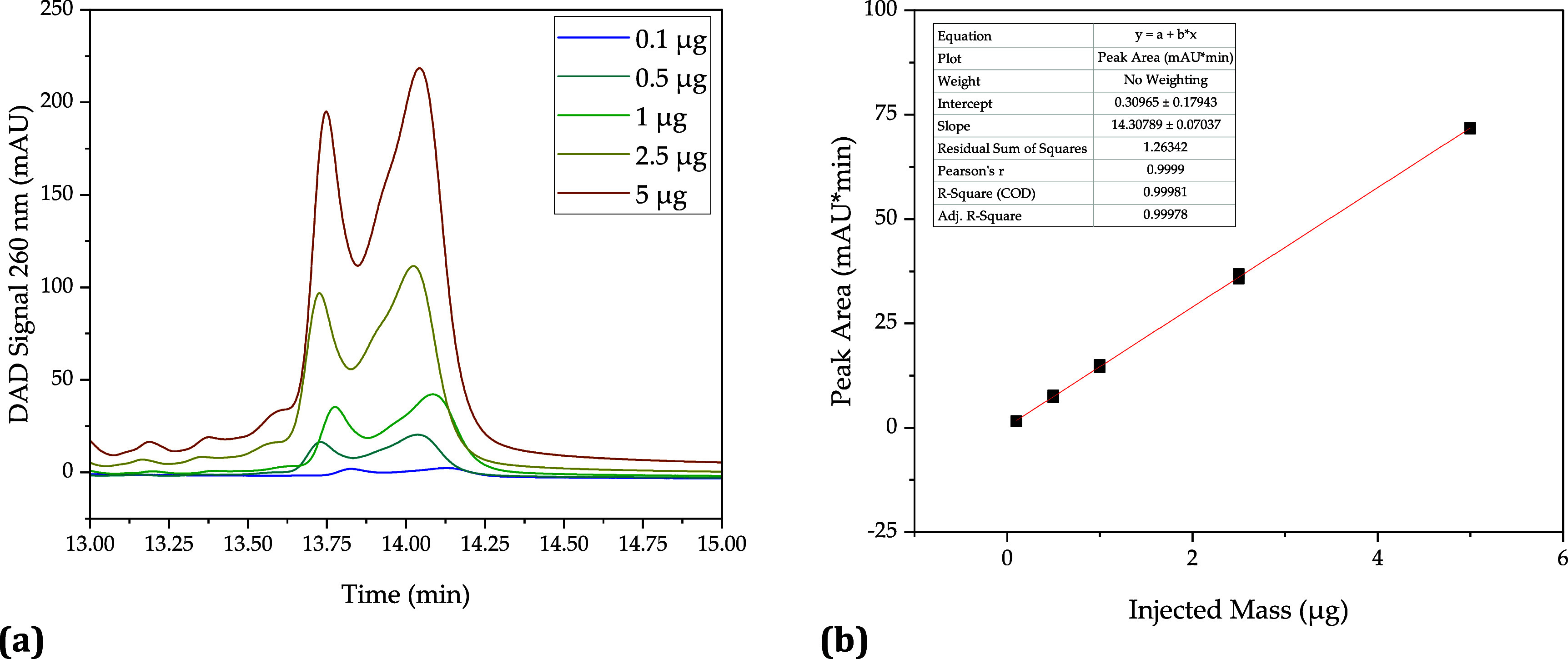
Calibration of the mRNA concentration measured by anion exchange chromatography. Chromatogram with increasing injection mass (a) and calibration line (b).

In this study, the amount of mRNA generated was determined by using a strong anion exchanger. Calibration was done analogously to RP-HPLC. The chromatograms as well as the determined calibration line are shown in Figure 4. The calibration is also very accurate here with an *R*^2^ of 1 (Figure 4b), and the measurements are also well-reproducible. Unlike RP, however, no shift of the mRNA peak is observed here at higher injection volumes (Figure 4a). The mRNA peak consists of two nonbaseline-separated peaks representing different isoforms of mRNA.

**Figure 5:**
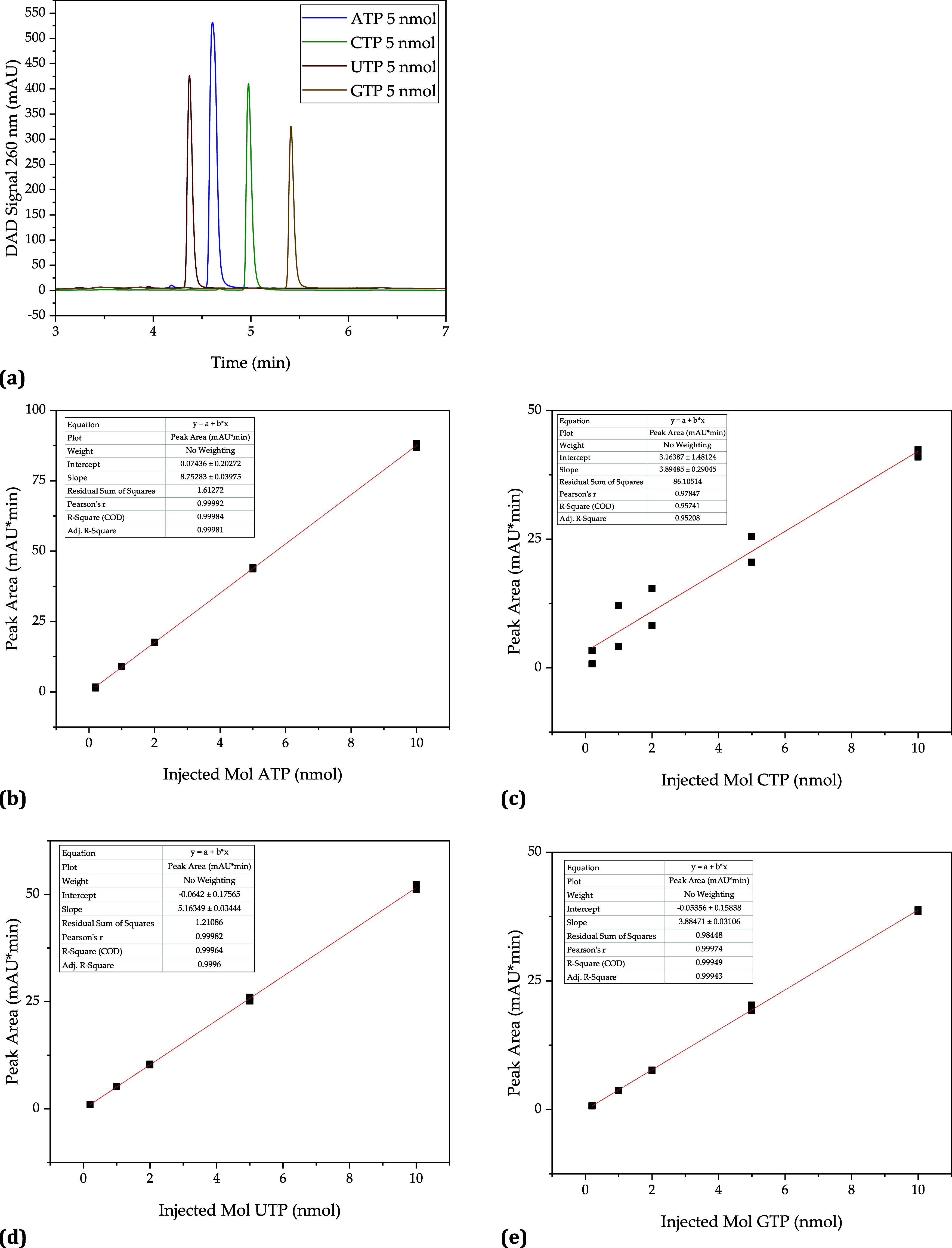
Calibration of the nucleotide concentration by anion exchange chromatography. Chromatogram showing baseline separation of ATP, CTP, GTP, and UTP (a), calibration line for ATP (b), calibration line for CTP (c), calibration line for UTP (d), and calibration line for GTP (e).

In addition to the mRNA, the nucleotides can also be separated via the anion exchanger (Figure 5a). As with the mRNA, five different concentration levels were selected and determined in duplicate to determine the calibration lines. For the nucleotides, these were 0.2, 1, 2, 5, and 10 nmol. The calibration of the nucleotides ATP (Figure 5a), UTP (Figure 5c), and GTP (Figure 5d) is very accurate with an *R*^2^ of 1, and the measurements are well-reproducible. For CTP (Figure 5b), on the other hand, *R*^2^ is slightly lower at 0.95. Nevertheless, the calibration is judged to be sufficiently accurate.

### 4.2 Kinetic Parameter Determination and Model Prediction

**Figure 6:**
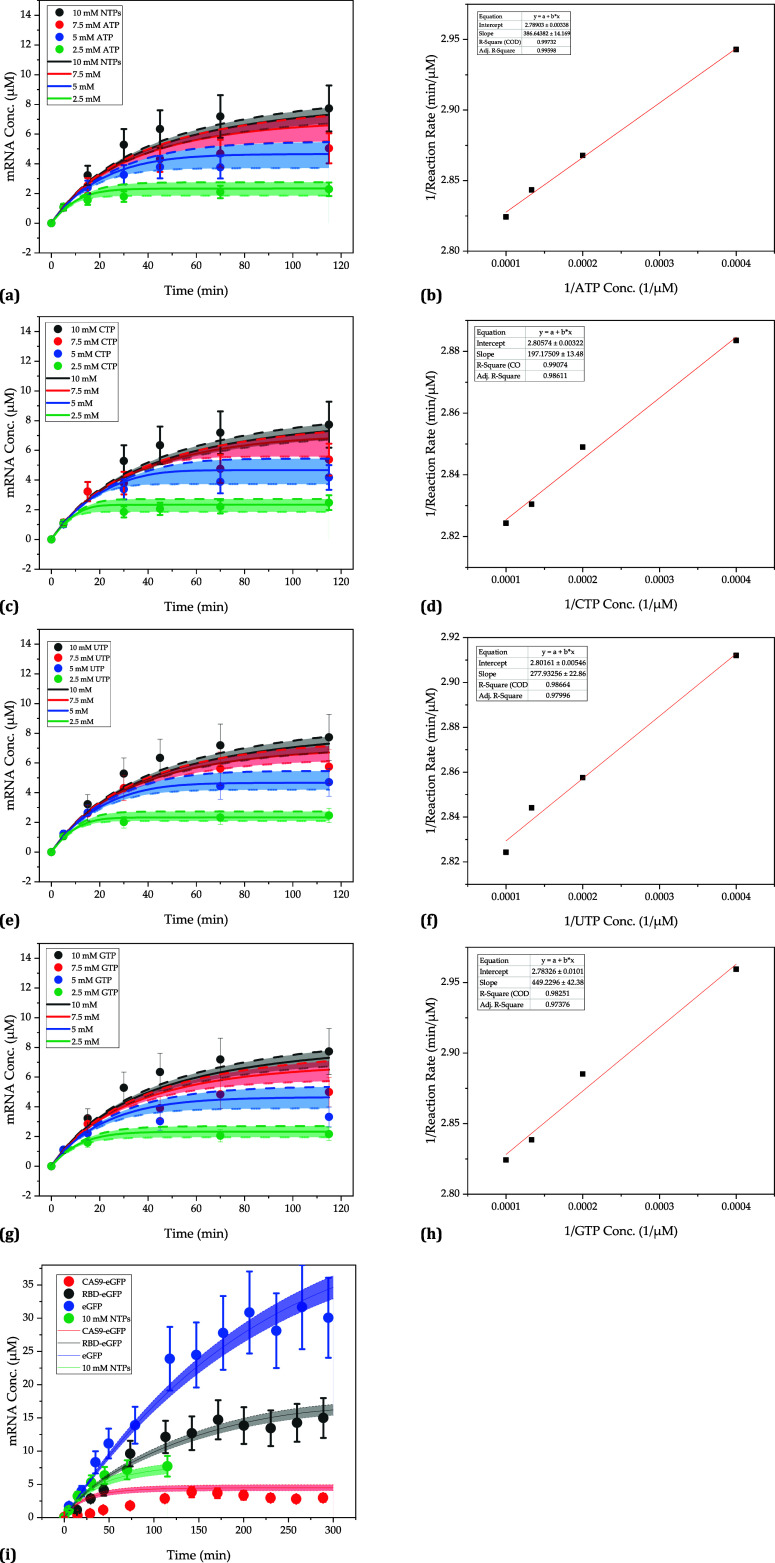
Experimental determination of Michaelis–Menten constants and Monte Carlo simulations for ATP (a), CTP (c), UTP (e), and GTP (g) and corresponding Lineweaver–Burk plots (b, d, f, and h). Validation of the model on an open-access independent data set from Rosa et al.^28^ with three alternative transcripts (i).

For the determination of the kinetic parameters, one nucleotide concentration was varied from 2.5 to 10 mM in each case, and the remaining nucleotides were used in excess. The remaining reaction conditions were kept at a nonlimiting stage as described in Section 3.3. Initial reaction rates are determined from the linear range at the beginning. The Michaelis–Menten constants as well as the maximum reaction rates were determined via linear regression of the Lineweaver–Burk (see Figure 6), Hanes–Woolf, and Eadie–Hofstee plots, as well as nonlinear regression of the Michaelis–Menten plot.

The regressions are sufficiently accurate with a minimum adj. *R*^2^ of 0.97. The maximum reaction rate is 0.358 ± 0.001 μM/min for all nucleotides. The Michaelis–Menten constants are 140.4 ± 2.9 μM for ATP, 71.5 ± 1.9 μM for CTP, 101.5 ± 3.8 μM for UTP, and 165.5 ± 6.4 μM for GTP. These agree well with literature values of 9.5 to 142 μM for ATP,^95−97^ 23 to 180 μM for CTP,^95,96,98^ 33 to 107 μM for UTP,^13,95,96^ and 76 to 234 μM for GTP.^13,95,96^ That GTP has a higher affinity constant than the other nucleotides is also consistent with previous studies^96,98,99^ and may be due to the role of GTP in initiating *in vitro* transcription.^96,99^ For the binding of NTP in the initiation step, an affinity constant of 600 μM^100^ and one of 41 μM^22^ in elongation have been reported. Accordingly, the Michaelis–Menten constant for GTP should lie between these two values.^13^

To show the applicability and validity of the determined kinetic parameters, they have been implemented in the model described in Section 3.3. The shaded areas were generated using 30 Monte Carlo simulations (for each experiment). Here, the precision of the model parameter determination and its significance on the prediction are represented by the randomized combination of the model parameter values within their determination precision. The experimentally generated data are within the ranges predicted by the simulation; thereby, the error of the simulations (width of the shaded areas) is comparable or smaller to the experimental error. Thus, it satisfies the criteria proposed by Sixt et al.^101^ for the validation of process models. Moreover, following Braatz et al.,^80^ an independent data set from Rosa et al.,^28^ which was not used to determine the kinetic parameters, was reproduced with the model (Figure 6i). It should be emphasized that not only the sequence but additionally the transcript length differs from that in this study. Agreement between the experimental and simulated values underlines the applicability of the model as a digital twin.

**Figure 7:**
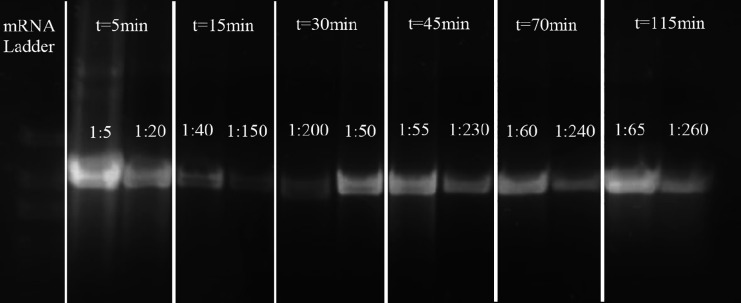
Progression of *in vitro* transcription resolved on agarose gel electrophoresis; NTP concentration of 10 mM, 6 time points, each with two dilutions indicated by the text.

The increasing concentration of mRNA with an increasing time can also be qualitatively described with agarose gel electrophoresis. The obtained gel image for 10 mM of all nucleotides is shown in Figure 7. Dilutions were adjusted according to the expected concentration curve published by Rosa et al., whose IVT was performed at similar conditions such that approximately 1 and 0.25 μg of mRNA were loaded onto the gel.^28^

### 4.3 High-Throughput Screening of Reaction Parameters in Continuous *In Vitro* Transcription

The reaction conditions have already been optimized for the batchwise production of mRNA. In addition, it has been shown that the continuous and scalable production of mRNA is feasible by generating a segmented flow and that comparable yields can be achieved as in the batchwise mode.^8^

Since supplementation of urea has already been shown in the literature to reduce dsRNA during *in vitro* transcription,^34^ in the previously mentioned study, urea was supplemented during the continuous *in vitro* transcription. However, this resulted in a 42% decrease in yield and a significant increase from 51 to 82% of truncated mRNA in the continuous production. Consequently, a full factorial experimental design comprising 15 experimental points including three center points was designed to optimize the reaction conditions in terms of yield and the proportion of fully formed mRNA. For this purpose, the temperature, urea concentration and reaction-enhancing additives were varied and statistically evaluated by means of stepwise reduction of the *p*-value.

**Figure 8:**
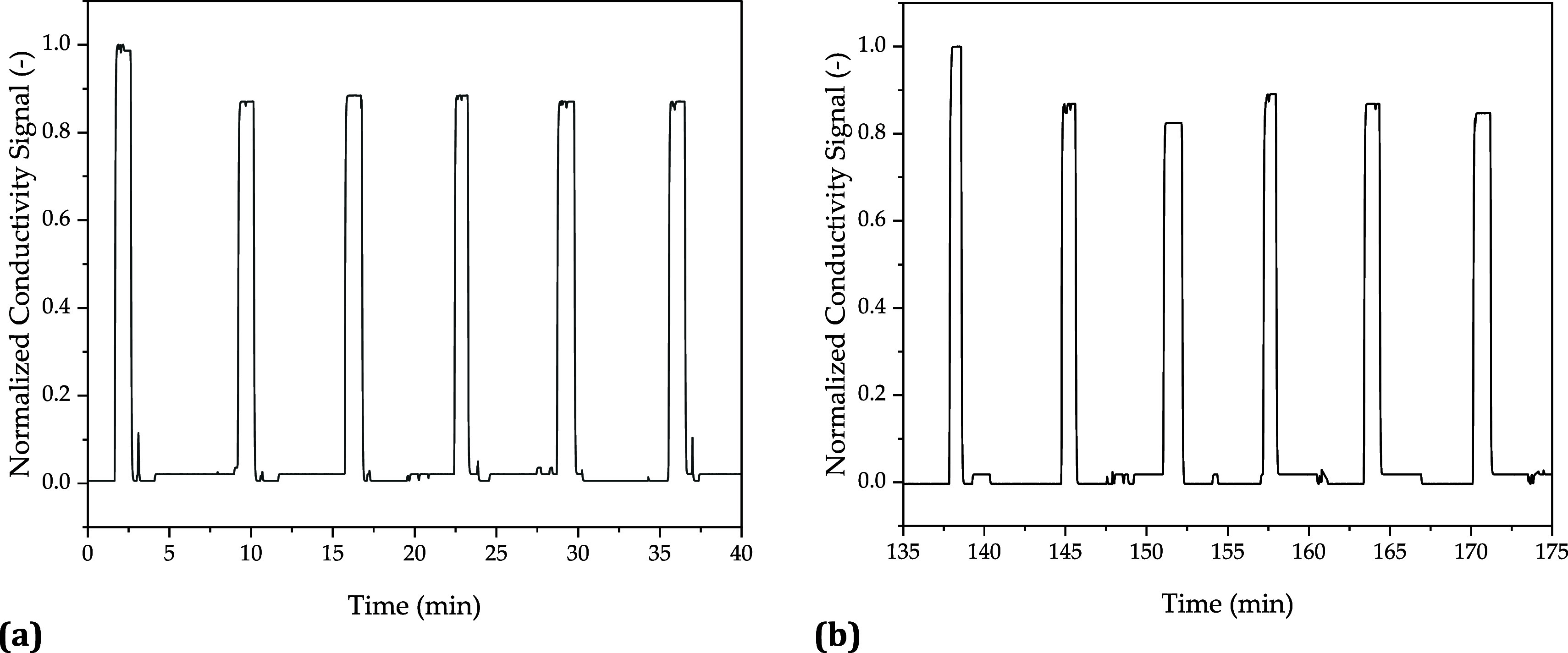
Input (a) and output (b) signals (conductivity) for six experimental points of the DoE.

The experiments were performed in the scalable mRNA machine described in the literature.^8^ By generating a segmented flow, the experimental points with the same temperature could be carried out directly one after the other. Slugs were detected using conductivity measurements. Signal-based fractionation is thereby possible with a product loss of <1% and contamination by the oil phase of <2%.^8^ For six experimental points of the DoE, which were successively brought into the plug flow reactor, the input (Figure 8a) and output (Figure 8b) signals are shown in Figure 8. The slugs have a mean residence time of 135 ± 0.6 min, with the distance between the individual injections differing by 0.3 ± 0.2 min comparing the input to the output signal. These deviations are due to fluctuations in the volume flow rate caused by fluctuations in the pump speed. By applying a control strategy based on PID controllers and validated process models as demonstrated by Schmidt et al., these fluctuations can be compensated.^9^

**Figure 9:**
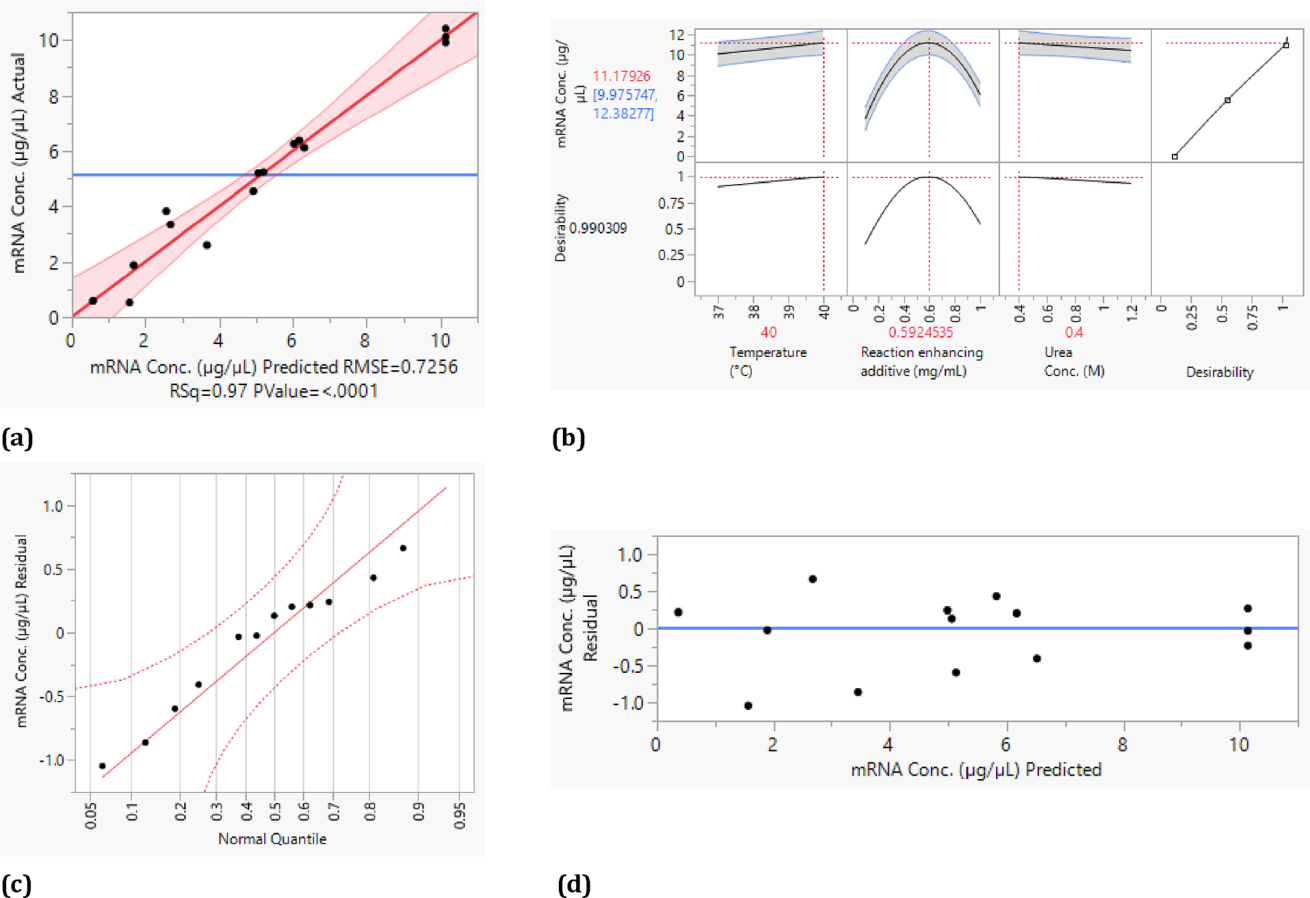
Statistical evaluation of the experiments. Actual vs predicted plot for mRNA concentration (a), profile plot (b), normal quantile plot (c), and residual plot (d).

**Figure 10:**
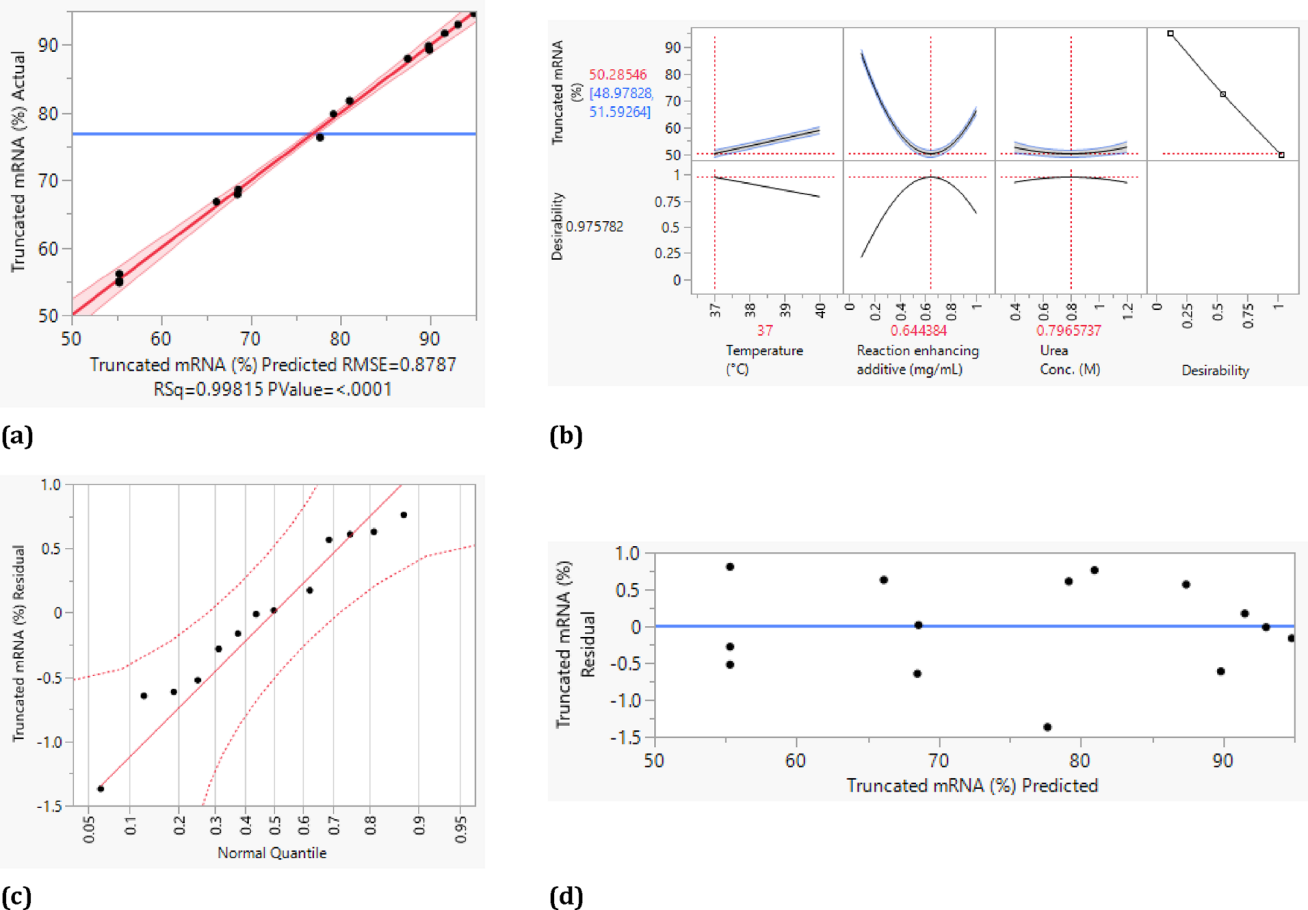
Statistical evaluation of the experiments. Actual vs predicted plot for truncated mRNA percentage (a), profile plot (b), normal quantile plot (c), and residual plot (d).

The results of the statistical evaluation with respect to mRNA concentration are shown in Figure 9, and those with respect to truncated mRNA are shown in Figure 10. The actual versus predicted plot (Figure 9a) indicates the quality of the statistical analysis. This is sufficiently accurate with an adj. *R*^2^ of 0.95. In addition, the *p*-value was reduced stepwise in the evaluation so that the results are statistically relevant and cannot be attributed to random chance. The residuals of the experiments were also used to evaluate the model quality. Figure 9d suggests homoscedasticity because the residuals are randomly distributed in a nearly constant-width band around the identity line. In addition, a normal distribution of the error terms can be assumed because the normal probability diagram of the residuals is almost linear (Figure 9c). For a maximum mRNA concentration, the reaction-enhancing additives could be identified as the main influencing factor by statistical analysis of the experimental design. The main effect of the temperature, as in the case of batchwise optimization of yield, is not significant in the range investigated.^8^ Also, the shutdown of the enzyme at 40 °C and 1.2 M urea observed in the literature^34^ was not observed in the continuous IVT with the addition of the reaction-enhancing additives. However, by maximizing the desirability (Figure 9b), we could observe the tendency to higher mRNA concentrations at higher temperatures. In addition, an optimal concentration of reaction-enhancing additives of 0.6 mg/mL was identified. The main effect of urea concentration is not significant in the range investigated, but there is a tendency for lower urea concentrations to lead to higher yields.

The statistical analysis with the truncated mRNA as the target variable also shows a sufficiently high quality with an adj. *R*^2^ of 0.996 and the stepwise reduction of the *p*-value (see Figure 10a). Similar to the mRNA yield, homoscedasticity can be assumed due to the random distribution of the residuals around the identity line (Figure 10d). Furthermore, the error terms in the normal probability diagram of the residuals (Figure 10d) lie almost on a straight line, which suggests a normal distribution of the error terms. The reaction-enhancing additives and their quadratic effects with themselves can also be identified as significant effects. In addition, after statistical evaluation, the interaction of the reaction-enhancing additives with temperature and the quadratic interaction of the urea concentration are significant. Desirability was maximized for a minimal amount of truncated mRNA (Figure 10c). Again, the optimal concentration of reaction-enhancing additives is approximately 0.6 mg/mL. The urea concentration, on the other hand, should be approximately 0.8 M, and the temperature should be 37 °C.

**Figure 11:**
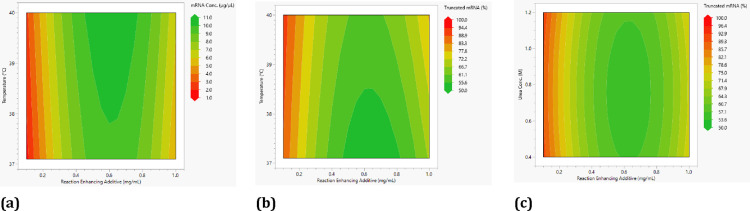
Contour plots showing the effect of the reaction-enhancing additive concentration and temperature on mRNA concentration (a) as well as for percent truncated mRNA (b). Effect of the urea concentration and reaction-enhancing additive concentration of percent truncated mRNA is shown on the right (c).

From the statistical analysis of the experimental design, the contour plots shown in Figure 11 can be generated. These also reflect that for a maximum yield (Figure 11a) and a minimum amount of truncated mRNA (Figure 11b,c), the concentration of the reaction-enhancing additives should be around 0.6 mg/mL. In addition, for the highest possible percentage of fully formed mRNA, the urea concentration should be around 0.8 M. As from the statistical analysis, it is also clear from the contour plots that high mRNA concentrations are obtained at higher temperatures, and lower proportions of truncated mRNA are obtained at lower temperatures. Thus, for the highest possible yield with a minimal amount of truncated mRNA, the temperature should be in the range of approximately 37.8 to 38.5 °C, as this is where the areas in the contour plots overlap with the most optimal process conditions for both target values. Consequently, the center point performed in this study with 0.55 mg/mL reaction-enhancing additives, 0.8 M urea, and a temperature of 38.5 °C is already very close to the optimum.

**Figure 12:**
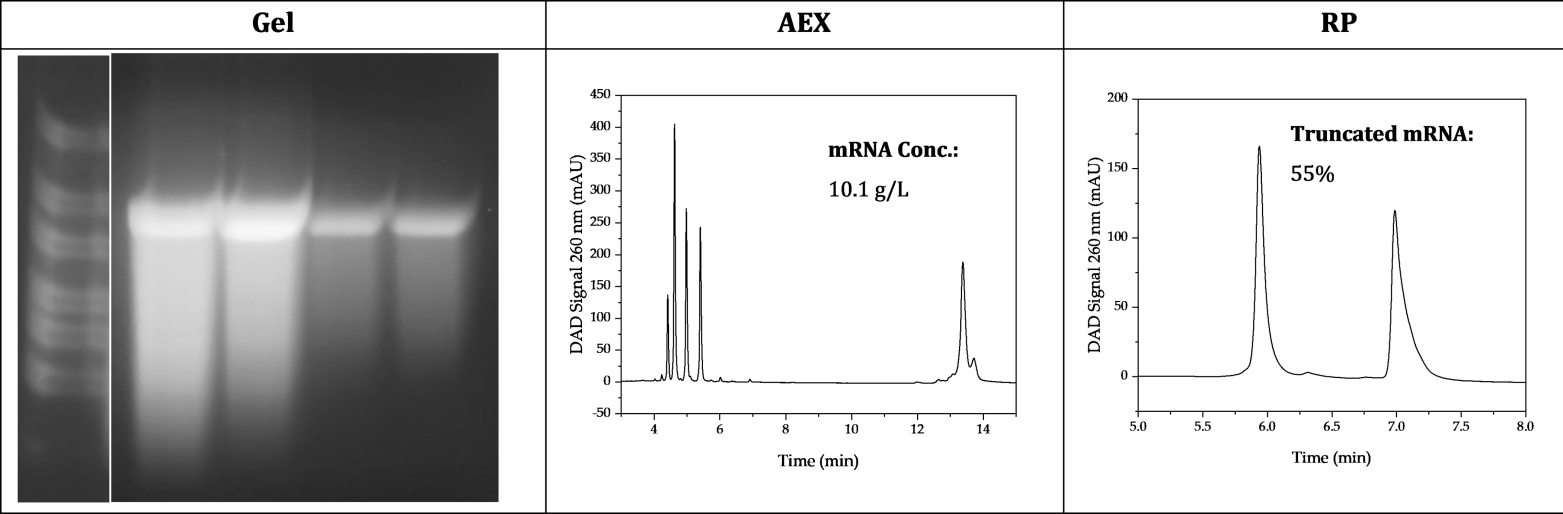
Agarose gel electrophoresis image (left), anion exchange (mid), and ion-pair reversed-phase (right) chromatogram of the optimal operating point.

At the optimal operating point, 10.1 ± 0.2 g/L mRNA with a fraction of 55.3 ± 0.6% truncated mRNA is generated (see Figure 12). As a result, the yield was increased by 55% compared to the starting point, and the amount of truncated mRNA was reduced by 33%. Thus, the yield is only about 1 g/L lower and the amount of truncated mRNA only 4% higher than the mRNA generated in the PFR without the addition of urea. In comparison, at less favorable process conditions such as 0.1 mg/mL reaction-enhancing additives, 1.2 M urea, and 37 °C, only 0.6 g/L mRNA is generated, of which 89% is truncated mRNA.

At the optimum operating point, approximately 74 ± 1% of all nucleotides are thus consumed. The consumption rates of all nucleotides are listed in Table 1. The highest consumption occurs for UTP and the lowest for GTP. ATP and CTP, on the other hand, are consumed almost equally.

**Table 1:** NTP Consumption for the Experimental Triplicates (CP1, CP2, and CP3) as well as mRNA Concentration Yield and Percent Truncated mRNA

	mRNA conc. (g/L)	truncated mRNA (%)	ATP consumption (%)	CTP consumption (%)	UTP consumption (%)	GTP consumption (%)
CP1	9.9	56.1	71.5	72.5	85.7	64.3
CP2	10.4	55.0	76.1	75.0	88.1	66.9
CP3	10.1	54.8	75.8	74.4	85.5	66.1

The reproduction of experimental points in the performance of high-throughput screening experiments by generating a segmented flow in a plug flow reactor can be performed with a deviation of 2% in the yield and 1.4% in the fraction of truncated RNA with sufficient accuracy. During the formation of truncated mRNA, it is not unusual for uneven nucleotide consumption to occur.^102^ However, it is striking that UTP is consumed the most, although it occurs the least in the sequence. One reason for this could be an unfavorable interaction with the organic phase compared to that of the other nucleotides.

## 5 Discussion and Conclusions

This study, in addition to determining the kinetic parameters for the process model of IVT, demonstrates the high-throughput screening on the same scalable mRNA machine presented in our earlier work.^8^

The analytical methods used in this study were IP-RP chromatography to determine the amount of truncated mRNA and strong anion exchange chromatography to determine the mRNA concentration. Both could be calibrated very accurately with an *R*^2^ close to 1 for the quantification of mRNA and for the determination of NTP concentrations.

The kinetic parameters could be determined that are needed for DT-based process optimization and automation.^103^ The maximum reaction rate is 0.358 ± 0.001 μM/min for all nucleotides. The second important parameter for modeling the reaction in the process model is the Michaelis–Menten constant, which is 140.4 ± 2.9 μM for ATP, 71.5 ± 1.9 μM for CTP, 101.5 ± 3.8 μM for UTP, and 165.5 ± 6.4 μM for GTP. These agree well with the values published in the literature.^13,95−98^ The experimentally determined kinetic parameters are suitable for model-based prediction reactions, which are substrate-limited. Furthermore, the model can be applied to other transcripts with different lengths and sequences. This illustrates the applicability of the model used in this study and the experimentally determined kinetic parameters as a basis for implementation as a digital twin in an automated process.

The supplementation of urea into the reaction mix serves to reduce dsRNA. However, in continuous production in the PFR, this results in a yield loss of up to 42% and a 56% increase in the amount of truncated mRNA. In this study, the reaction conditions were optimized in terms of temperature, urea concentration, and reaction-enhancing additives concentration, resulting in a yield of 10.1 g/L with 55.3% truncated mRNA. Thus, the yield was increased by 55% compared to the starting point, and the amount of truncated mRNA was reduced by 33%. To maximize the yield of mRNA, previous studies have already shown that the nucleotide concentration and magnesium ion concentration have the greatest influence on concentration,^12,28^ which was already optimized in batch and applied to the continuous production.^8^ Based on the observations, the temperature in combination with a high urea concentration can lead to a total shutdown of the polymerase.^34^ Furthermore, possible interactions of the temperature with the reaction-enhancing additive should be investigated. For maximum yield, a concentration of 0.6 mg/mL of the most influencing factor, the reaction-enhancing additives, is optimal. Furthermore, the shutdown of the enzyme at 40 °C and 1.2 M urea observed^34^ was not observed in the continuous IVT with the addition of the reaction-enhancing additives. To minimize the amount of truncated mRNA, 0.6 mg/mL reaction-enhancing additives are also optimal. Additionally, the urea concentration is significant and should be 0.8 M. In contrast to the yield, increasing the temperature results in less fully developed mRNA. Consequently, the temperature should be in the range of approximately 37.8 to 38.5 °C based on the contour plots of the statistical analysis of the DoE.

To speed up the execution of the experimental design in the PFR, the experiments were performed as a segmented flow. The distance between the generated slugs shifted by an average of only 18 s at a mean residence time of 135 min. The deviations can be compensated by automating the process by integrating PAT and digital twins, as already shown for mRNA and pDNA production.^9,104^ The good reproducibility with a maximum deviation of 2% can thus extend the advantages of the scalable machine.^8^ In this study, it could be shown that in addition to the application of the mRNA machine for all phases of vaccine approval, from 1,000 clinical doses up to 10 million manufacturing scale doses, it is also possible to produce many possible vaccine candidates in a short time in only one machine. The different slugs were fed in an interval of approximately 6.6 min. Consequently, by applying high-throughput screening through the generation of a segmented flow, instead of two vaccine candidates, 20 vaccine candidates can be produced within 270 min, which means an increase in productivity by a factor of 10.

In summary, with detailed process comprehension of the IVT fundamentals, the conditions for the operation of continuous *in vitro* transcription could be optimized in this work to produce 55% more mRNA with 33% less truncated mRNA, compared to our initial starting point.^8^ To our knowledge, this is the first publication of performing DoE-supported IVT optimization continuously in segmented flow.

The results published here are the basis for a fully continuous, bottleneck-free production process of mRNA, including HTS, which can in the future be adapted to other drugs/vaccine candidates. For this, a predictive process model and process analytical technologies, as well as the continuous formulation of mRNA into lipid nanoparticles as described in detail already,^105^ will be needed. Potential drug candidates can be screened in the PFR by generating a segmented flow in a high-throughput approach and then manufactured from 1,000 clinical doses to the 10 million manufacturing scale doses in one GMP- and QbD-compliant Biopharma 4.0 facility, enabled by the integration of state-of-the-art PAT and predictive validated process models.
